# CD90 serves as differential modulator of subcutaneous and visceral adipose-derived stem cells by regulating AKT activation that influences adipose tissue and metabolic homeostasis

**DOI:** 10.1186/s13287-019-1459-7

**Published:** 2019-11-28

**Authors:** Zhenzhen Pan, Zixin Zhou, Huiying Zhang, Hui Zhao, Peixuan Song, Di Wang, Jilong Yin, Wanyi Zhao, Zhaoxiang Xie, Fuwu Wang, Yan Li, Chun Guo, Faliang Zhu, Lining Zhang, Qun Wang

**Affiliations:** 1 Key Laboratory of Infection and Immunity of Shandong Province, Department of Immunology, School of Basic Medical Sciences, Shandong University, 44 Wenhua Xi Road, Jinan, 250012 Shandong People’s Republic of China; 2 Department of Clinical Laboratory, The Second Hospital of Shandong University, Jinan, 250033 Shandong People’s Republic of China; 3 School of Mathematics and Statistics, Shandong University, Weihai, 264209 Shandong People’s Republic of China; 4 Key Laboratory of the Ministry of Education for Experimental Teratology, Department of Histology and Embryology, School of Basic Medical Science, Shandong University, Jinan, 250012 Shandong People’s Republic of China; 5 Department of Pathogen Biology, School of Basic Medical Science, Shandong University, Jinan, 250012 Shandong People’s Republic of China

**Keywords:** CD90, Adipose-derived stem cell, Proliferation, Mitotic clonal expansion, Adipose tissue, Metabolic homeostasis

## Abstract

**Background:**

White adipose tissue includes subcutaneous and visceral adipose tissue (SAT and VAT) with different metabolic features. SAT protects from metabolic disorders, while VAT promotes them. The proliferative and adipogenic potentials of adipose-derived stem cells (ADSCs) are critical for maintaining adipose tissue homeostasis through driving adipocyte hyperplasia and inhibiting pathological hypertrophy. However, it remains to be elucidated the critical molecules that regulate different potentials of subcutaneous and visceral ADSCs (S-ADSCs, V-ADSCs) and mediate distinct metabolic properties of SAT and VAT. CD90 is a glycosylphosphatidylinositol-anchored protein on various cells, which is also expressed on ADSCs. However, its expression patterns and differential regulation on S-ADSCs and V-ADSCs remain unclear.

**Methods:**

S-ADSCs and V-ADSCs were detected for CD90 expression. Proliferation, colony formation, cell cycle, mitotic clonal expansion, and adipogenic differentiation were assayed in S-ADSCs, V-ADSCs, or *CD90*-silenced S-ADSCs. Glucose tolerance test and adipocyte hypertrophy were examined in mice after silencing of *CD90* in SAT. *CD90* expression and its association with *CyclinD1* and *Leptin* were analyzed in adipose tissue from mice and humans. Regulation of AKT by CD90 was detected using a co-transfection system.

**Results:**

Compared with V-ADSCs, S-ADSCs expressed high level of CD90 and showed increases in proliferation, mitotic clonal expansion, and adipogenic differentiation, together with AKT activation and G1-S phase transition. *CD90* silencing inhibited AKT activation and S phase entry, thereby curbing proliferation and mitotic clonal expansion of S-ADSCs. In vivo *CD90* silencing in SAT inhibited S-ADSC proliferation, which caused adipocyte hypertrophy and glucose intolerance in mice. Furthermore, *CD90* was highly expressed in SAT rather than in VAT in human and mouse, which had positive correlation with *CyclinD1* but negative correlation with *Leptin.* CD90 promoted AKT activation through recruiting its pleckstrin homology domain to plasma membrane.

**Conclusions:**

CD90 is differentially expressed on S-ADSCs and V-ADSCs, and plays critical roles in ADSC proliferation, mitotic clonal expansion, and hemostasis of adipose tissue and metabolism. These findings identify CD90 as a crucial modulator of S-ADSCs and V-ADSCs to mediate distinct metabolic features of SAT and VAT, thus proposing CD90 as a valuable biomarker or target for evaluating ADSC potentials, monitoring or treating obesity-associated metabolic disorders.

## Background

Excess fat accumulation in adipose tissue causes obesity, which increases the risks of diabetes, steatohepatitis, stroke, cardiovascular disease, or even cancer [1, 2]. White adipose tissue (WAT) including subcutaneous and visceral adipose tissue (SAT and VAT) stores surplus energy to regulate metabolic balance. Both SAT and VAT expand during adiposity but with distinct metabolic functions. The accumulation of VAT is associated with metabolic disorders including insulin resistance, diabetes mellitus, hypertension, dyslipidemia, and atherosclerosis, while SAT improves insulin action and metabolism [3–5]. Studies from humans and animal models provided evidences for the protective effects of SAT but adverse effects of VAT on metabolism. Removal of visceral fat in aging rats or omentectomy in obese subjects improved their metabolic profiles, while transplantation of subcutaneous fat into intra-abdominal site decreased body weight and fat mass, and improved insulin sensitivity in recipient mice [6–8].

WAT expands via adipocyte hyperplasia and hypertrophy, characterized by increases in number of new adipocytes or size of existing adipocytes, respectively [9–12]. Adipocyte hyperplasia indicates de novo adipogenesis from progenitors in response to metabolic demands. It has been demonstrated that hyperplasic expansion of SAT protected the mice against obesity-induced insulin resistance, whereas loss of hyperplasic potential caused pathological hypertrophic expansion of SAT that led to adipose tissue dysfunction, inflammation, and systemic insulin resistance in animals and humans [10, 13–15]. A recent study also substantiated that de novo adipocyte differentiation protected against pathologic visceral adipose expansion in obesity and resulted in improvements in glucose homeostasis [16], suggesting the beneficial roles of adipocyte hyperplasia in WAT and metabolic homeostasis. Currently, there are still some controversy over de novo adipogenesis in SAT and VAT [17]. Joe and colleagues concluded that SAT expanded mostly by hyperplasia, whereas VAT by hypertrophy, as SAT had more proliferating adipogenic progenitors than VAT in mice fed on high-fat diet [18]. Conversely, another study showed that VAT had higher capacity of adipogenesis by hyperplasia than SAT in response to high-fat diet feeding using an in vivo adipogenesis tracking mouse model, though the stromal vascular fraction (SVF) from SAT was easier to differentiate into adipocytes in vitro than that from VAT [19]. In addition, Macotela et al. reported that CD34- and SCA1-positive adipocyte precursor cells from SAT had higher capacity for adipogenic differentiation in vitro than those from VAT in mice [20]. So, the balance between adipocyte hyperplasia and hypertrophy in SAT and VAT especially related regulatory mechanisms remains to be clarified.

Adipose-derived stem cells (ADSCs) expressing mesenchymal stem cell (MSC) markers like CD44, CD105, CD90, and CD73 are the main progenitors in WAT, which can differentiate into multiple cell types in vitro including adipocytes, osteoblasts, and chondrocytes. Due to their capacities for self-renewal and multipotent differentiation, ADSCs play pivotal roles in supporting WAT homeostasis under pathophysiological conditions and have broad prospects in tissue repair and regeneration [21–25]. ADSCs are essential progenitors for adipocyte hyperplasia, which links cell proliferation with differentiation during adipogenic differentiation [12]. In the initial phase of adipogenesis, growth-arrested preadipocytes (ADSCs committed to adipocyte lineage) reenter cell cycle to undergo several rounds of cell division, known as mitotic clonal expansion, which is a prerequisite for adipogenic differentiation [12, 26–28]. Reagents blocking cell cycle reentry or proliferation significantly impaired adipocyte differentiation through inhibiting clonal expansion [12, 29, 30], confirming the proliferative potential of ADSCs in determining adipogenic differentiation. Considering the differences between SAT and VAT, key molecules and mechanisms for regulating the proliferative potentials and differences between subcutaneous and visceral ADSCs (S-ADSCs, V-ADSCs) require to be clarified.

CD90, also known as Thy-1, is a typical glycosylphosphatidylinositol (GPI)-anchored protein, which functions differently in various cells including regulating cell proliferation, apoptosis, survival, adhesion, and migration [31–34]. The effects of CD90 on cell proliferation have been studied in several types of tumor cells, fibroblasts, and hematopoietic stem cells, which either promotes or inhibits proliferation depending on different cell types in various contexts [32, 33, 35–37]. Several studies also showed the differential impacts of CD90 on differentiation of MSCs based on their different species or tissue sources [38–40]. As one of the MSC markers, CD90 is also expressed on ADSCs, but its expression patterns and differential impacts on S-ADSCs and V-ADSCs remain unclear. A recent study showed an important role of CD90 in AKT activation in human cytomegalovirus-infected cells [41]. AKT activation is involved in the survival or proliferation of various cells and can promote the proliferation of human and mice ADSCs by upregulating cell cycle protein CyclinD1 [42–46]. However, it remains largely unknown whether CD90 regulates ADSC potentials via AKT and whether CD90 produces different impacts on S-ADSCs and V-ADSCs to influence adipose tissue and metabolic homeostasis. In the present study, we demonstrated that CD90 had different expression profiles on S-ADSCs and V-ADSCs, which differentially regulated proliferation and mitotic clonal expansion of S-ADSCs and V-ADSCs through modulating AKT activation, thereby producing distinct impacts on WAT homeostasis and metabolism. This study proposes CD90 as a critical target for regulating ADSCs, which has potential prospects in therapy for obesity-associated metabolic disorders.

## Methods

### Animals

C57BL/6 male mice were provided by Vital River Laboratory Animal Technology Co. Ltd. (Beijing, China). All animal studies were approved by the Ethical Committee of Qilu Hospital of Shandong University, and all experimental procedures were performed in accordance with the institutional guidelines for animal care and utilization.

### Isolation and culture of ADSCs

S-ADSCs and V-ADSCs were isolated from inguinal and epididymal WAT of C57BL/6 male mice at the age of 10–12 weeks. Briefly, fat pads were digested with 2 mg/mL collagenase (Worthington, Lakewood, NJ) in Krebs-Ringer Bicarbonate buffer at 37 °C for about 50 min. The SVF passed through a 100-μm filter was incubated overnight in complete Dulbecco’s modified Eagle’s medium (DMEM) containing 10% fetal bovine serum (Invitrogen, Carlsbad, CA) and 5 ng/mL basic fibroblast growth factor (Peprotech, Rocky Hill, NJ). After removal of non-adherent cells, the adherent cells were cultured as ADSCs. The third to fifth passages were used for the experiments.

### Assay for proliferation, mitotic clonal expansion, colony formation, and cell cycle

The growth curve of ADSCs was examined using Cell Counting Kit (CCK)-8 (Dojindo, Tokyo, Japan), and optical density (OD) value was measured at 450 nm to evaluate viability of the cells. For EdU incorporation assay, ADSCs were cultured in 96-well plates at 37 °C, 5%CO_2_ overnight, and then were incubated with EdU (10 μM) for additional 6 h before harvest. EdU incorporation was detected by Cell-Light EdU Apollo567 Cell Tracking Kit (RiboBio, Guangzhou, China) according to the manufacturer’s instruction. Mitotic clonal expansion was examined in ADSCs subjected to 16 h of adipogenic induction by EdU incorporation assay. For colony formation assay, ADSCs were cultured in 6-well plates at 37 °C, 5% CO_2_ for 7 days, and then were stained with crystal violet for colony counting after fixed with 100% methanol. For cell cycle assay, ADSCs were fixed with ice-cold 70% ethanol at 4 °C overnight and then incubated with propidium iodide (PI) at 4 °C for 30 min. The cells were acquired and analyzed with Cytomics FC500 (Beckman Coulter, Pasadena, CA). In some experiments, S-ADSCs were transfected with CD90 siRNA (siCD90, Sigma-Aldrich, San Francisco, CA) using Jet-PRIME (Polyplus, Berkeley, CA) and then used for the above assays. The sequences of siCD90 were listed (Additional file 1: Table S1).

### Adipogenic differentiation

ADSCs were cultured to over confluence and further induced using adipogenic differentiation medium (Cyagen Biosciences, Guangzhou, China) according to the manufacturer’s instruction. The adipogenic differentiation was evaluated by Oil Red O staining (Sigma-Aldrich) after 18 days of induction, and OD value was measured at 500 nm after eluting Oil Red O with 100% isopropanol. In some experiments, S-ADSCs infected with shRNA CD90 (shCD90) or control shRNA (shControl) recombinant lentivirus (GenePharma, shanghai, China) were subjected to adipogenic induction. The sequences of shCD90 were listed (Additional file 1: Table S1).

### Flow cytometry

ADSCs were incubated with Fc block and then stained with fluorescein isothiocyanate (FITC)-labeled antibody (Ab) against mouse CD90 (clone: 30-H12), phycoerythrin (PE)-labeled Ab against mouse CD105 (clone: MU7/18), and PE-cyanine5 (PE-cy5)-labeled Ab against mouse CD44 (clone: IM7) (eBioscience, San Diego, CA). The cells were acquired using CytoFLEX S, and data were analyzed by CytExpert (Beckman Coulter).

### In vivo lentiviral infection

Male mice at the age of 8 weeks were anesthetized, and the bilateral inguinal fat pads were exposed and injected with shCD90 or shControl lentivirus (Genechem, Shanghai, China) (Additional file 1: Table S1). The mice were maintained under standard conditions after surgery, and glucose tolerance test (GTT) was conducted 3 weeks later. Briefly, mice were fasted overnight and blood glucose levels were determined at different time points after intraperitoneal injection of glucose (2 g/kg body weight, Sigma-Aldrich). Four weeks later, inguinal adipose tissue was collected from the mice. The average sizes of adipocytes were measured in adipose tissue section after hematoxylin and eosin (H&E) staining using Image-Pro Plus 6.0. ADSCs isolated from inguinal adipose tissue were examined for CD90 expression and proliferative potential.

### Database analysis

*CD90* expression and its correlation with *CyclinD1* and *Leptin* were analyzed in mice and human adipose tissue using GEO databases. The following databases were included in the study: (1) gene expression profiles of inguinal and axillary SAT, and epididymal and mesenteric VAT from age-matched C57BL/6 male mice fed on normal diet (GSE53307); (2) gene expression profiles of epididymal and mesenteric VAT from C57BL/6 mice fed on normal or high-fat diet for 2, 4, 8, 20, and 24 weeks (GSE39549); (3) gene expression of epididymal VAT including adipocyte and stromal vascular cell (SVC) fractions from male C57BL/6 mice fed on normal or high-fat diet for 0, 3, and 7 days (GSE65557); (4) gene expression of abdominal SAT from subjects (body mass index, BMI, 16.7–50.2) with normal or impaired glucose tolerance, or type 2 diabetes (GSE27951); (5) gene expression of SAT and omental VAT from BMI-matched, morbidly obese patients who were insulin sensitive or resistant (GSE15773); and (6) gene expression of SAT and omental VAT from BMI-matched, obese patients who were insulin sensitive or resistant (GSE20950).

### Plasmid transfection and immunofluorescence

Plasmids carrying genes encoding human active pleckstrin homology (PH) domain of AKT (pcDNA3-AKT-PH-GFP) or mutant AKT-PH domain (pcDNA3-AKT-PH^R25C^-GFP) were kindly provided by Dr. Craig Montell from Johns Hopkins University via addgene (Cambridge, MA) [47]. Plasmids pENTER (Mock) and pENTER-THY1(CD90)-Flag were purchased from ViGene BioScieneces (Jinan, China). HEK-293T cells planted in 24-well chamber slides were co-transfected with pcDNA3-AKT-PH-GFP (or pcDNA3-AKT-PH^R25C^-GFP) and pENTER-CD90-Flag (or Mock) for 24 h. After fixed in 4% paraformaldehyde for 30 min and blocked with 5% bovine serum albumin (BSA) for 1 h, the cells were incubated with anti-Flag (DDDDK) Ab (MBL, Woburn, MA) at 4 °C overnight, followed by incubation with Alexa Fluor 594-conjucted secondary Ab (Proteintech Group, Chicago, IL) at 37 °C for 1 h. The nuclei were stained with 4,6-diamidino-2-phenylindole (Beyotime Biotechnology, Shanghai, China). Fluorescent signals were analyzed with laser scanning confocal microscope (Zeiss, Jena, Germany).

### Quantitative PCR

Total RNA was extracted from cells or tissues using RNAfast200 (Fastagen, Shanghai, China) or Trizol (TIANGEN BIOTECH, Beijing, China), and reversely transcripted into cDNA with ReverTra Ace qPCR RT Kit (TOYOBO Life Science, Shanghai, China). qPCR was carried out using SYBR Green Master Mix (CWbiotech, Beijing, China). The relative mRNA levels of interested genes were evaluated using 2^-△△Ct^ method, using *18 s* rRNA or *GAPDH* as internal control. The primers were listed (Additional file 1: Table S2).

### Western blot

Equal amounts of proteins from cell or tissue lysates were loaded on SDS-PAGE gels. After electrophoresis, proteins were transferred to PVDF membranes. After blocked with 5% BSA for 3 h, the membranes were blotted with Abs against mouse AKT, phosphor (p)-AKT, CyclinD1 (Cell Signaling Technology, Beverly, MA), CD90 (Biolegend, San Diego, CA), GAPDH, or Tubulin (Proteintech Group) at 4 °C overnight, followed by incubation with HRP-conjugated secondary Ab (ZSGB-BIO, Beijing, China) for 1 h. The signals were detected by SuperSignal West Pico Chemiluminescent Substrate (Pierce Biotechnology, Rockford, IL).

### Statistical analysis

Data were expressed as mean ± SEM. Statistical differences were evaluated using Student’s *t* test, one- or two-way ANOVA, or non-parametric test, respectively. *P* < 0.05 was considered significant.

## Results

### S-ADSCs show higher potential in proliferation than V-ADSCs through promoting AKT activation

**Fig. 1:**
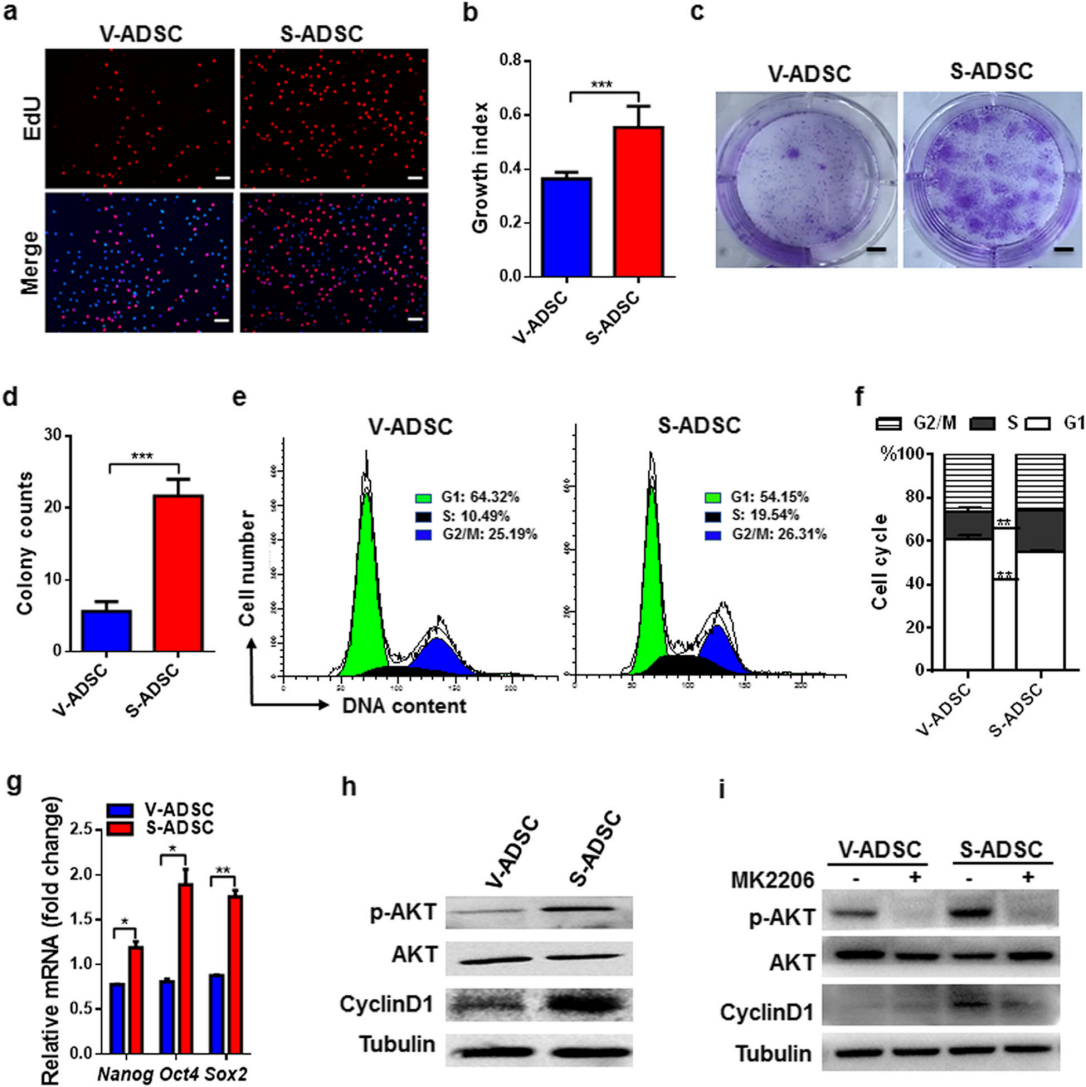
S-ADSCs show higher potential in proliferation than V-ADSCs through promoting AKT activation. S-ADSCs and V-ADSCs from inguinal and epididymal adipose tissue of the mice (*n* = 10) were used in the following experiments. **a**, **b** Cell proliferation was determined by EdU incorporation assay. Fluorescence signals were examined by fluorescence microscope, and growth index (number of EdU-positive nuclei/number of all nuclei) was calculated. Representative (**a**) and statistic (**b**) data are shown. Scale bar 100 μm. **c**, **d** Colony formation was measured by crystal violet staining. Representative (**c**) and statistic (**d**) data are shown. Scale bar 8.5 mm. **e**, **f** Cell cycle profiles were analyzed by flow cytometry after PI staining. Representative (**e**) and statistic (**f**) data are shown. **g** The expression of stemness genes was measured by qPCR. **h**, **i** Protein levels of p-AKT, AKT, and CyclinD1 were detected on S-ADSCs and V-ADSCs in the absence (**h**) or presence (**i**) of MK2206 (2 μM) for 20 h by western blot. Data are presented as mean ± SEM. *n* = 3–5 per group. *P < 0.05, **P < 0.01, ***P < 0.001

The proliferation of S-ADSCs and V-ADSCs was examined by EdU incorporation assay. Compared with V-ADSCs, S-ADSCs showed significant increase in EdU-positive cells (red in nuclear indicates cells in S phase), suggesting that S-ADSCs proliferate more rapidly than V-ADSCs (Fig. 1a, b). Accordingly, S-ADSCs formed more clones than V-ADSCs in colony formation assay (Fig. 1c, d). Cell cycle profiles showed that S-ADSCs had a marked decrease in G1 phase but increase in S phase compared to V-ADSCs (Fig. 1e, f). The expression levels of stemness genes *Nanog*, *Oct4*, and *Sox2* were markedly upregulated in S-ADSCs compared with those in V-ADSCs (Fig. 1g). Importantly, S-ADSCs showed significant increase in AKT phosphorylation compared with V-ADSCs, together with CyclinD1 upregulation that drives G1-S phase transition (Fig. 1h). While in the presence of AKT inhibitor MK2206, the upregulation of CyclinD1 in S-ADSCs was obviously impaired after blockade of AKT phosphorylation (Fig. 1i). These data suggest that AKT activation may contribute to effective proliferation of S-ADSCs through driving G1-S phase transition.

### S-ADSCs show higher potential in adipogenic differentiation than V-ADSCs through promoting mitotic clonal expansion

**Fig. 2:**
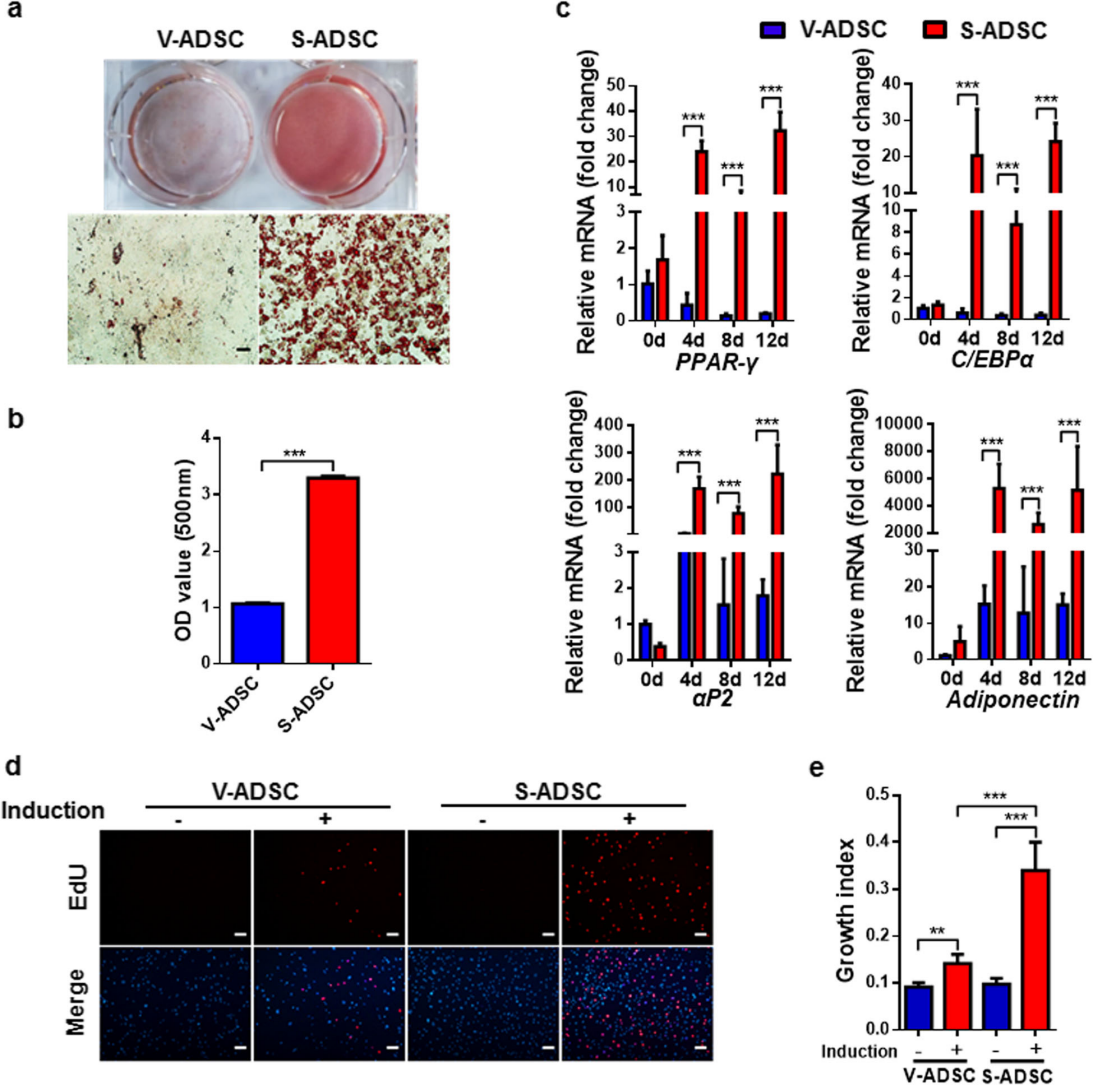
S-ADSCs show higher potential in adipogenic differentiation than V-ADSCs through promoting mitotic clonal expansion. S-ADSCs and V-ADSCs prepared as specified in Fig. 1 were subjected to adipogenic induction. **a**, **b** After 18 days of adipogenic induction, lipid contents were visualized using Oil Red O staining (**a**) and quantified by elution of Oil Red O (**b**). Scale bar 50 μm. **c** Gene expression of white adipocyte markers was measured by qPCR at days 0, 4, 8, and 12 after adipogenic induction. **d**, **e** Mitotic clonal expansion was determined by EdU incorporation assay after 16 h of adipogenic induction. Fluorescence signals were examined by fluorescence microscope (**d**). Scale bar 100 μm. Growth indices as specified in Fig. 1 are shown (**e**). Data are presented as mean ± SEM. *n* = 4–6 per group. **P < 0.01, ***P < 0.001

Considering the differences in proliferation and stemness between S-ADSCs and V-ADSCs, the adipogenic differentiation between S-ADSCs and V-ADSCs was further compared. After 18 days of adipogenic induction, S-ADSCs were fully differentiated into adipocytes with numerous lipid droplets, while V-ADSCs showed poor ability to differentiate into adipocytes, with only a few lipid droplets in them. These observations were further confirmed by quantification of lipid contents (Fig. 2a, b). Correspondingly, the mRNA levels of white adipocyte markers *PPAR-γ*, *C/EBPα*, *αP2*, and *Adiponectin* were dramatically increased in S-ADSCs after 4, 8, or 12 days of adipogenic induction, which were significantly higher than those in differentiating V-ADSCs (Fig. 2c). These data indicate that S-ADSCs have higher adipogenic potential than V-ADSCs.

Since the initial events determining adipogenic efficiency are cell cycle reentry and mitotic clonal expansion, we further compared the mitotic clonal expansion between S-ADSCs and V-ADSCs in the early stage of adipogenic differentiation. Both S-ADSCs and V-ADSCs showed obvious growth arrest before adipogenic induction. After 16 h of adipogenic induction, EdU-positive cells were observed in both S-ADSCs and V-ADSCs. Differently, S-ADSCs had a significant increase in EdU-positive cells compared with V-ADSCs upon adipogenic induction, suggesting that S-ADSCs had higher capacity for S phase entry and mitotic clonal expansion than V-ADSCs in response to adipogenic induction (Fig. 2d, e).

### CD90 is highly expressed on S-ADSCs rather than V-ADSCs

**Fig. 3:**
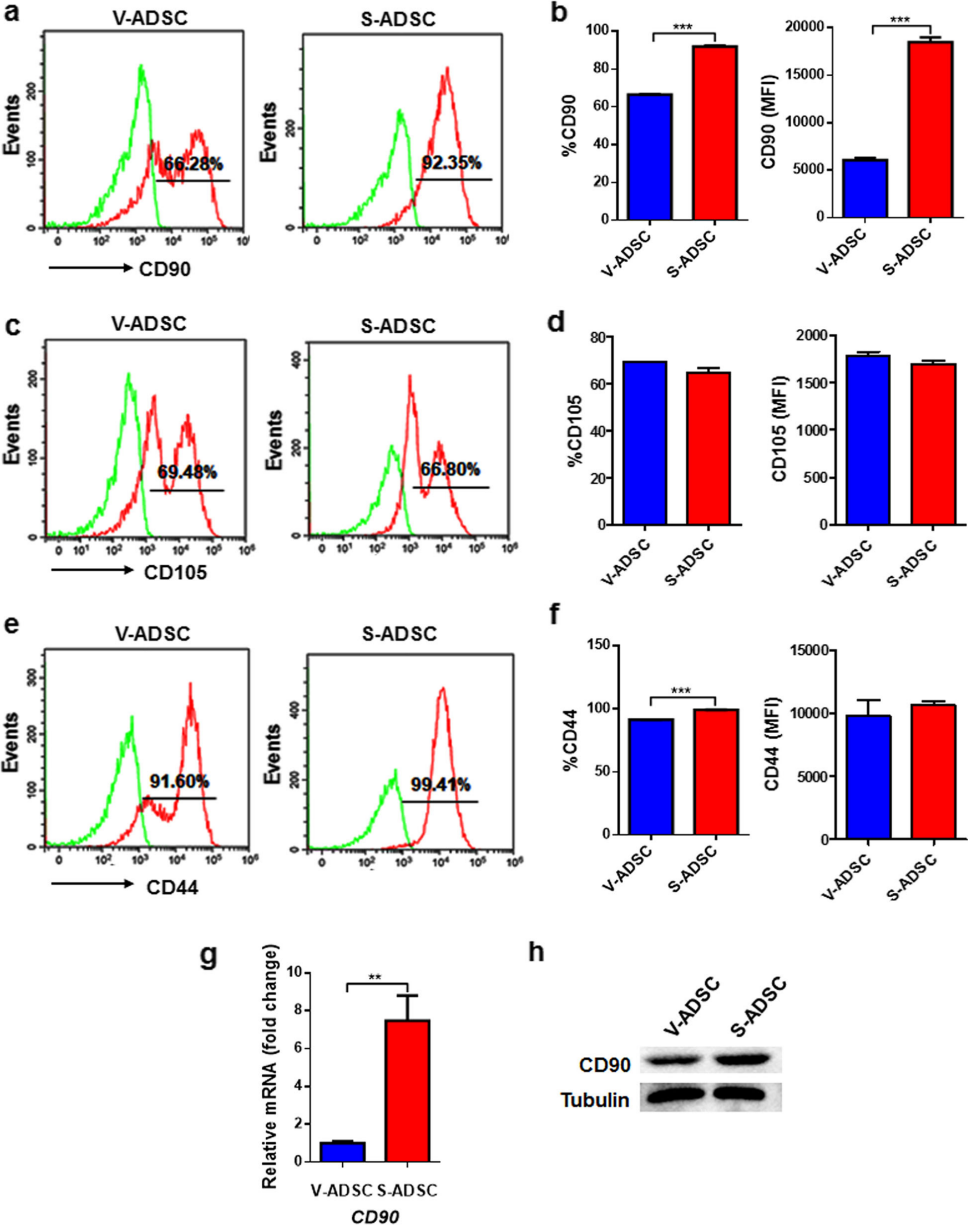
CD90 is expressed highly on S-ADSCs rather than V-ADSCs. S-ADSCs and V-ADSCs prepared as specified in Fig. 1 were examined for stem cell-related markers. **a**–**f** The expression of CD90, CD105, and CD44 was detected by flow cytometry. Representative (**a**, **c**, **e**) and statistic (**b**, **d**, **f**) data are shown. **g**, **h** The mRNA and protein levels of CD90 were analyzed by qPCR (**g**) and western blot (**h**). Data are presented as mean ± SEM. *n* = 3–6 per group. **P < 0.01, ***P < 0.001

To clarify the possible reasons for differences in proliferation and mitotic clonal expansion between S-ADSCs and V-ADSCs, we examined the expression profiles of stem cell-related markers on ADSCs. As expected, both S-ADSCs and V-ADSCs positively expressed CD90, CD105, and CD44. Differently, S-ADSCs expressed higher level (both percentage and intensity) of CD90 than V-ADSCs (Fig. 3a, b), while no obvious differences were observed in CD105 expression between S-ADSCs and V-ADSCs (Fig. 3c, d); though there is a slight increase in CD44 percentage on S-ADSCs compared with V-ADSCs, both of them express high levels of CD44 (Fig. 3e, f). Consistently, high levels of CD90 mRNA and protein in S-ADSCs rather than in V-ADSCs were confirmed by qPCR and western blot (Fig. 3g, h). These findings demonstrate that S-ADSCs and V-ADSCs have different expression profiles of CD90, which may serve as an important modulator of their distinct biological behaviors.

### *CD90* silencing inhibits proliferation of S-ADSCs by attenuating AKT activation

**Fig. 4:**
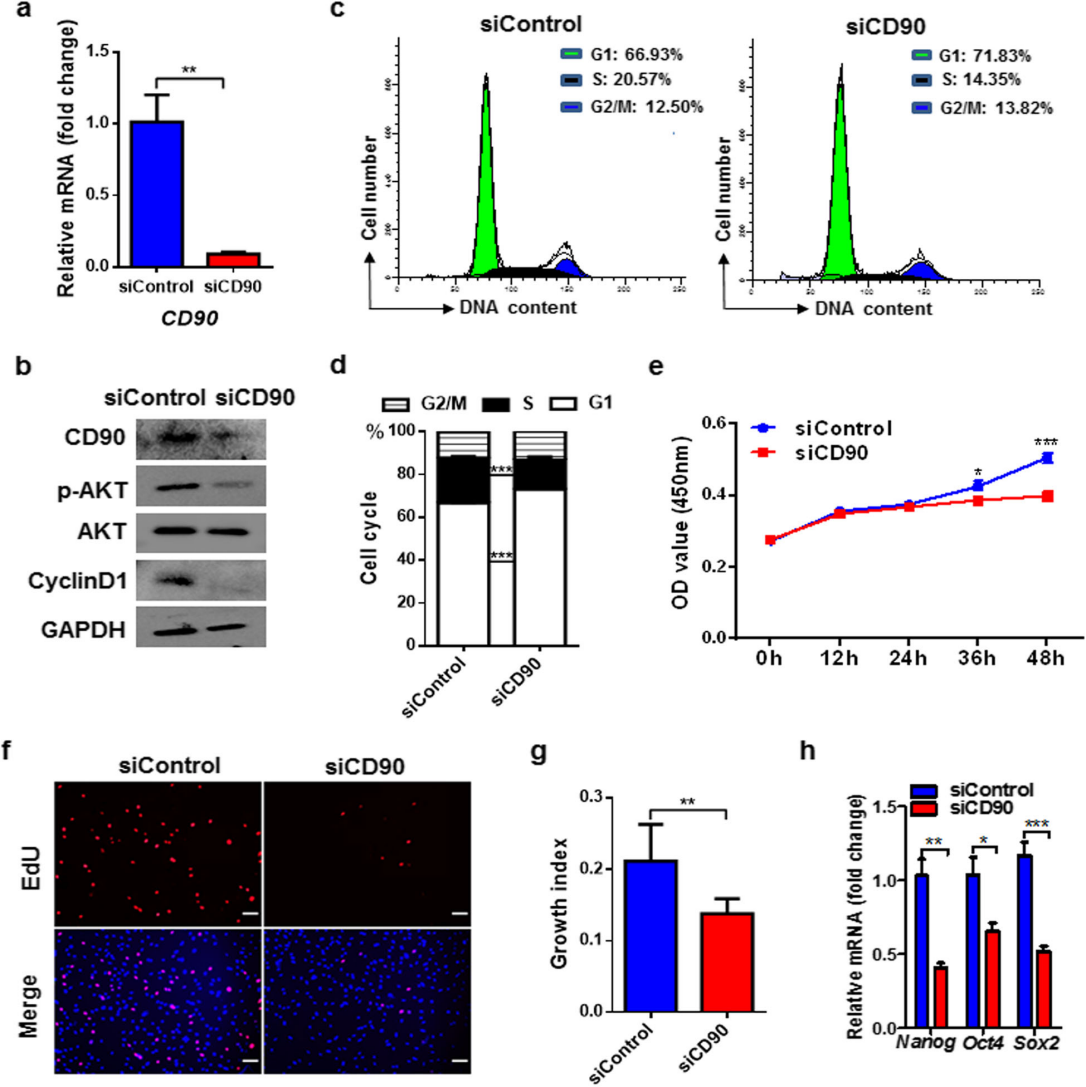
*CD90* silencing inhibits proliferation of S-ADSCs by attenuating AKT activation. S-ADSCs from mice (*n* = 10) were transfected with siCD90 or siControl for 24 h and then were collected for assays. **a** The mRNA level of *CD90* was detected by qPCR. **b** Protein levels of CD90, p-AKT, AKT, and CyclinD1 were detected by western blot. **c**, **d** Cell cycle profile was analyzed by flow cytometry after PI staining; typical (**c**) and statistic (**d**) data are shown. **e** Grow curve was determined by CCK-8 assay (*n* = 15 per condition). **f**, **g** Cell proliferation was determined by EdU incorporation assay. Fluorescence signals were examined by fluorescence microscope (**f**). Scale bar 100 μm. Growth indices as specified in Fig. 1 are shown (**g**). **h** The mRNA levels of stemness markers were measured by qPCR. Data are presented as mean ± SEM. *n* = 3–6 per group. *P < 0.05, **P < 0.01, ***P < 0.001

To explore the roles of CD90 in proliferation of S-ADSCs, we knocked down the expression of CD90 in S-ADSCs and found marked reductions in AKT phosphorylation and CyclinD1 expression (Fig. 4a, b). Different from the rapid response on AKT phosphorylation in the control group, insulin failed to induce the increase of AKT phosphorylation in *CD90*-silenced S-ADSCs, confirming that CD90 exerts an indispensable role in AKT activation in S-ADSCs (Additional file 1: Figure S1). Accordingly, *CD90* silencing caused significant G1 phase arrest together with S phase inhibition in S-ADSCs (Fig. 4c, d). Consequently, *CD90*-silenced S-ADSCs showed obviously reduced proliferation as suggested by decreases in growth curve and EdU-positive cells (Fig. 4e–g). Consistently, significant decreases in the mRNA levels of *Nanog*, *Oct4*, and *Sox2* were observed in *CD90*-silenced S-ADSCs (Fig. 4h). These findings indicate that CD90 promotes ADSC proliferation and stemness through activating AKT pathway and driving CyclinD1-mediated G1-S phase transition.

### *CD90* silencing inhibits mitotic clonal expansion of S-ADSCs that influences adipocyte differentiation

**Fig. 5:**
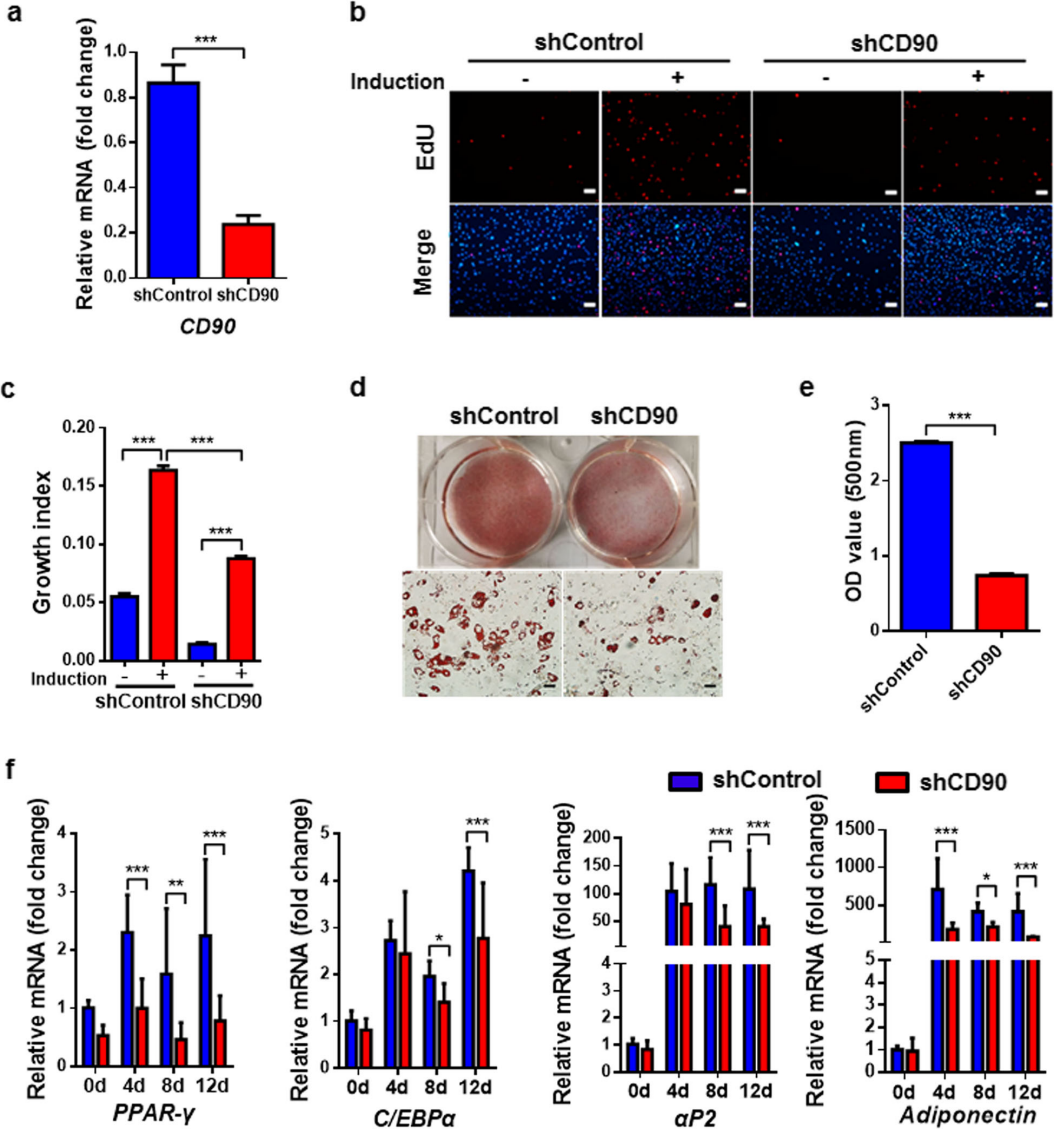
*CD90* silencing inhibits mitotic clonal expansion of S-ADSCs to influence adipocyte differentiation. S-ADSCs from mice (*n* = 10) were infected with shCD90 or shControl lentivirus for 72 h and then were subjected to adipogenic induction. **a** The mRNA level of CD90 was detected by qPCR before adipogenic induction. **b**, **c** Mitotic clonal expansion was determined by EdU incorporation assay after 16 h of adipogenic induction. Fluorescence signals were detected by fluorescence microscope (**b**). Scale bar 100 μm. Growth indices as specified in Fig. 1 are shown (**c**). **d**, **e** After 18 days of adipogenic induction, lipid contents were visualized using Oil Red O staining (**d**) and quantified by eluting Oil Red O (**e**). Scale bar 50 μm. **f** The mRNA levels of white adipocyte markers were measured by qPCR at days 0, 4, 8, and 12 during the induction. Data are presented as mean ± SEM. *n* = 4–6 per group. *P < 0.05, **P < 0.01, ***P < 0.001

Next, S-ADSCs were efficiently infected with GFP-tagged shCD90 or shControl lentivirus (Additional file 1: Figure S2). Compared with shControl, shCD90 led to a marked reduction in *CD90* expression in S-ADSCs (Fig. 5a). Although adipogenic induction caused increase of EdU-positive cells in both shControl- and shCD90-treated S-ADSCs, there were much less EdU-positive cells in shCD90-treated S-ADSCs than those in shControl-treated S-ADSCs, indicating that *CD90* silencing obviously inhibits mitotic clonal expansion and cell division of S-ADSCs during the early phase of adipogenic differentiation (Fig. 5b, c). Accordingly, after 18 days of adipogenic induction, shCD90-treated S-ADSCs showed obvious reduction in lipid droplets compared with shControl-treated S-ADSCs, which was verified by quantification of lipid contents (Fig. 5d, e). Meanwhile, shCD90 markedly inhibited the mRNA levels of white adipocyte markers *PPAR-γ*, *C/EBPα*, *αP2*, and *Adiponectin* in differentiating S-ADSCs (Fig. 5f). These findings suggest that CD90 promotes the mitotic clonal expansion of S-ADSCs, thereby facilitating their adipogenic differentiation.

### *CD90* silencing causes glucose intolerance and adipocyte hypertrophy in mice

**Fig. 6:**
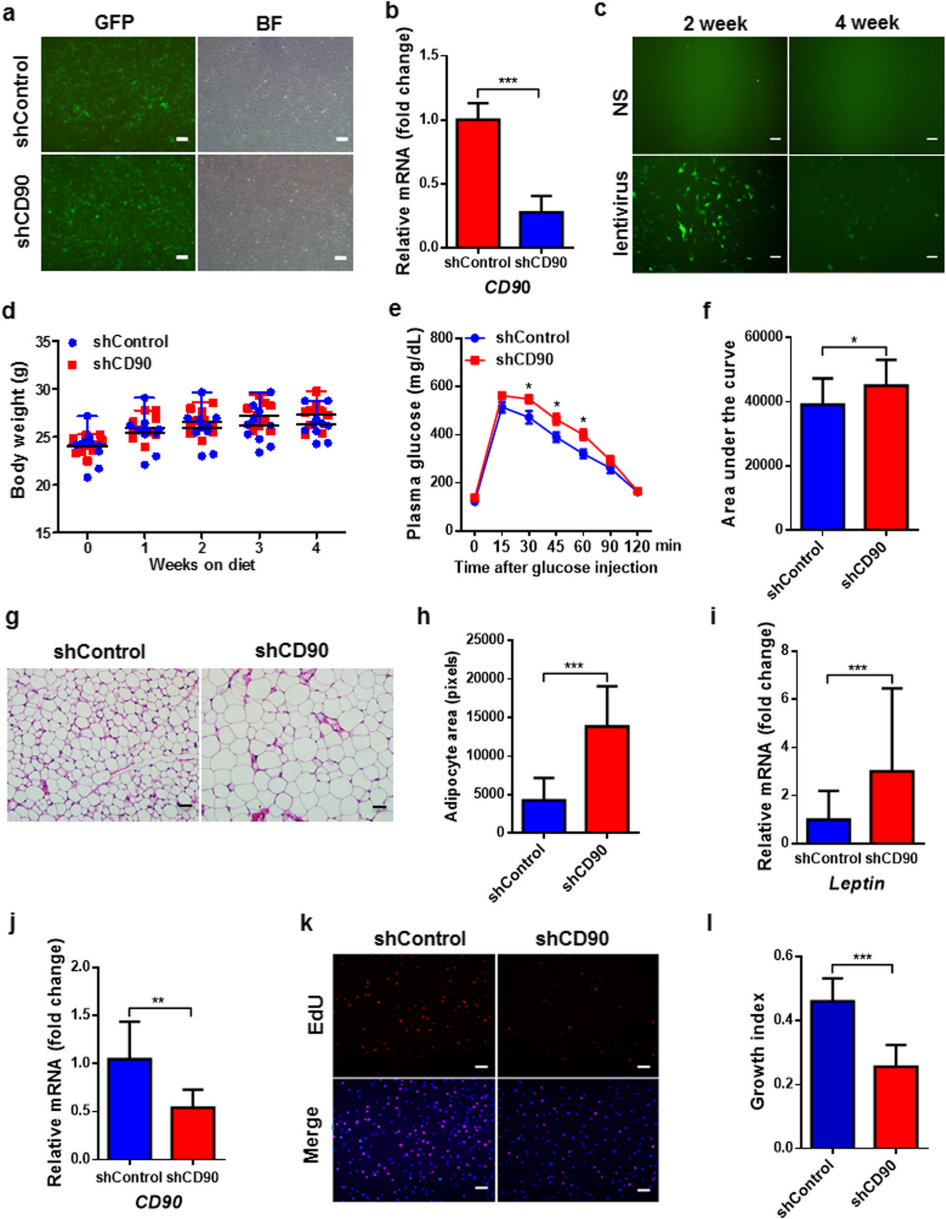
*CD90* silencing causes glucose intolerance and adipocyte hypertrophy in mice. **a**, **b** S-ADSCs were infected with GFP-tagged shControl or shCD90 lentivirus (Genechem). The infection efficiency was determined by GFP signals under fluorescence microscope (**a**). BF, bright field. Scale bar = 100 μm. The mRNA level of CD90 was detected by qPCR (**b**). **c** GFP-tagged shControl lentivirus was injected into bilateral inguinal fat pads (7.0 × 10^6^ TU per point, 3 points per fat pad) of male mice at the age of 8 weeks. After 2 or 4 weeks, ADSCs were isolated from inguinal SAT to examine GFP signals under fluorescence microscope. Scale bar = 50 μm. NS, normal saline. **d**–**l** Mice (*n* = 10 per group) at the age of 8 weeks were injected with shCD90 (or shControl) lentivirus as mentioned above. The body weight (**d**) was recorded during 4 weeks of treatment. After 3 weeks of treatment, GTT was performed in mice with overnight fast; glucose levels (**e**) were determined at different time points after glucose injection. Areas under the curve (**f**) for GTT are shown. The inguinal SAT section was stained with H&E (**g**), and the areas of adipocytes were measured as pixies using Image-Pro Plus 6.0 (**h**). Scale bar = 50 μm. The mRNA level of *Leptin* in inguinal SAT was detected by qPCR (**i**). S-ADSCs from inguinal SAT were examined for the mRNA level of *CD90* by qPCR (**j**) and proliferation by EDU incorporation assay. Fluorescence signals (**k**) detected by fluorescence microscope and growth indices (**l**) as specified in Fig. 1 are shown. Scale bar = 100 μm. Bars represent mean ± SEM. *P < 0.05, **P < 0.01, ***P < 0.001

To evaluate the influence of CD90 on WAT and systemic metabolism, we knocked down *CD90* in inguinal SAT of the mice using recombinant lentivirus. After determining the efficiencies of lentiviral infection and *CD90* silencing by shCD90 in S-ADSCs in vitro (Fig. 6a, b), the efficiency of lentiviral infection was examined in vivo*.* The results showed that GFP signals were clearly observed in inguinal S-ADSCs from the mice 2 weeks after injection with lentivirus, which lasted until 4 weeks (Fig. 6c). Compared with control mice, shCD90-treated mice showed no significant alterations in body weight and WAT weight till the end of the experiment (Fig. 6d and Additional file 1: Figure S3). However, shCD90 treatment led to a significant glucose intolerance in mice, as evidenced by difficulty in decreasing glucose levels at 30, 45, and 60 min after glucose injection and elevation in area under the curve during GTT (Fig. 6e, f). Importantly, shCD90-treated mice showed a marked increase in adipocyte size in SAT compared with shControl-treated mice (Fig. 6g, h). The mRNA level of *Leptin*, which has been proved to be positively correlated with adipocyte size [48], was also elevated in this fat depot from shCD90-treated mice (Fig. 6i). As expected, S-ADSCs from shCD90-treated mice, which had reduced expression of *CD90*, showed significant decrease in proliferation compared with those from shControl-treated mice (Fig. 6j–l). These data suggest that *CD90* silencing in SAT results in adipocyte hypertrophy and glucose intolerance in mice through suppressing ADSC proliferation.

### *CD90* is positively correlated with *CyclinD1* but negatively with *Leptin* in mouse and human WAT

**Fig. 7:**
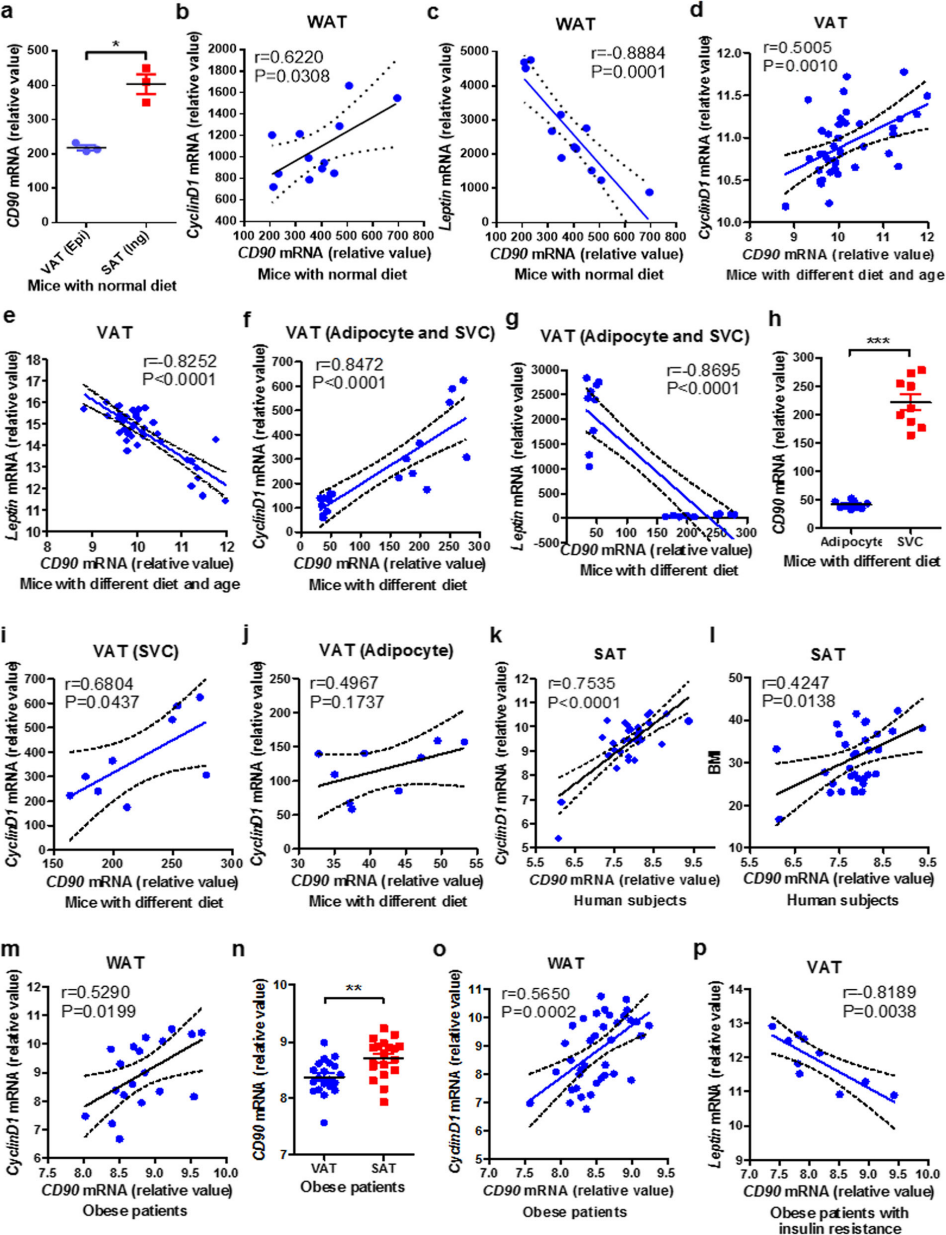
*CD90* is positively correlated with *CyclinD1* but negatively with *Leptin* in mouse and human WAT. **a**–**c** The expression of *CD90* (**a**) was analyzed in inguinal SAT and epididymal VAT from mice fed on normal diet (GSE53307, *n* = 12); the correlations of *CD90* expression with *CyclinD1* (**b**) and *Leptin* (**c**) expression were analyzed in WAT (inguinal, axillary SAT and epididymal, mesenteric VAT). **d**, **e** The correlations of *CD90* expression with *CyclinD1* (**d**) and *Leptin* (**e**) expression were analyzed in epididymal and mesenteric VAT from mice fed on normal or high-fat diet for 2, 4, 8, 20, and 24 weeks (GSE39549, *n* = 40). **f**–**j** The correlations of *CD90* expression with *CyclinD1* (**f**) and *Leptin* (**g**) expression were analyzed in epididymal VAT (adipocyte and SVC fractions) from mice fed on short term of normal or high-fat diet (GSE65557, *n* = 18); the expression of *CD90* (**h**) and its correlations with *CyclinD1* were analyzed on SVCs (**i**) and adipocytes (**j**). **k**, **l** The correlations of *CD90* expression with *CyclinD1* expression (**k**) and BMI (**l**) were analyzed in abdominal SAT from human subjects with normal or impaired glucose tolerance, or type 2 diabetes (GSE27951, *n* = 33). **m** The correlation of *CD90* expression with *CyclinD1* expression was analyzed in SAT and omental VAT from obese patients who were insulin sensitive or resistant (GSE15773, *n* = 19). **n**–**p** The expression of *CD90* (**n**) and its correlation with *CyclinD1* (**o**) were analyzed in WAT (SAT and omental VAT) from obese patients (GSE20950, *n* = 39); the correlation of *CD90* with *Leptin* (**p**) was analyzed in omental VAT from obese patients with insulin resistance. *P < 0.05, **P < 0.01, ***P < 0.001

To further verify the effects of CD90 on WAT homeostasis, we analyzed the mRNA level of *CD90* in mouse and human WAT using GEO database. In mice fed on normal diet, *CD90* was highly expressed in inguinal SAT compared with that in epididymal VAT, which was consistent with the expression profiles on S-ADSCs and V-ADSCs. *CD90* expression was positively correlated to proliferation-related *CyclinD1* expression, but negatively correlated to adipocyte hypertrophy-related *Leptin* expression in WAT (inguinal, axillary SAT and epididymal, mesenteric VAT) from these mice (Fig. 7a–c and Additional file 1: Figure S4A–C). A highly positive correlation of *CD90* with *CyclinD1* but a negative correlation with *Leptin* were also observed in epididymal and mesenteric VAT from mice fed on normal or high-fat diet for different time periods (Fig. 7d, e). Similarly, the correlations of *CD90* with *CyclinD1* or *Leptin* were found in epididymal VAT (adipocytes and SVCs) from mice fed on short term of normal or high-fat diet (Fig. 7f, g and Additional file 1: Figure S4D, E). It should be noted that *CD90* is highly expressed on SVCs that contained amounts of ADSCs, rather than on adipocytes (Fig. 7h). *CD90* on SVCs but not on adipocytes had a positive correlation with *CyclinD1* (Fig. 7i, j), indicating the predominant expression of CD90 on ADSCs is the primary contributor to CyclinD1 regulation and WAT homeostasis.

In human subjects with normal or impaired glucose tolerance, or type 2 diabetes, *CD90* expression in abdominal SAT was positively correlated with *CyclinD1* expression, as well as the BMI of the subjects (Fig. 7k, l)*.* The positive correlation of *CD90* with *CyclinD1* was also found in WAT (SAT and omental VAT) from obese patients with insulin sensitivity or resistance (Fig. 7m)*.* In particular, results from obese patients who were insulin sensitive or resistant showed that *CD90* was highly expressed in SAT compared with that in omental VAT, which was positively correlated to *CyclinD1* expression (Fig. 7n, o and Additional file 1: Figure S4F). Especially in insulin-resistant obese patients, *CD90* expression had a tightly negative correlation with *Leptin* expression in VAT (Fig. 7p). These observations from human WAT further confirmed the positive regulation of CD90 on CyclinD1 and WAT homeostasis.

### CD90 recruits AKT-PH domain to plasma membrane to promote AKT activation

**Fig. 8:**
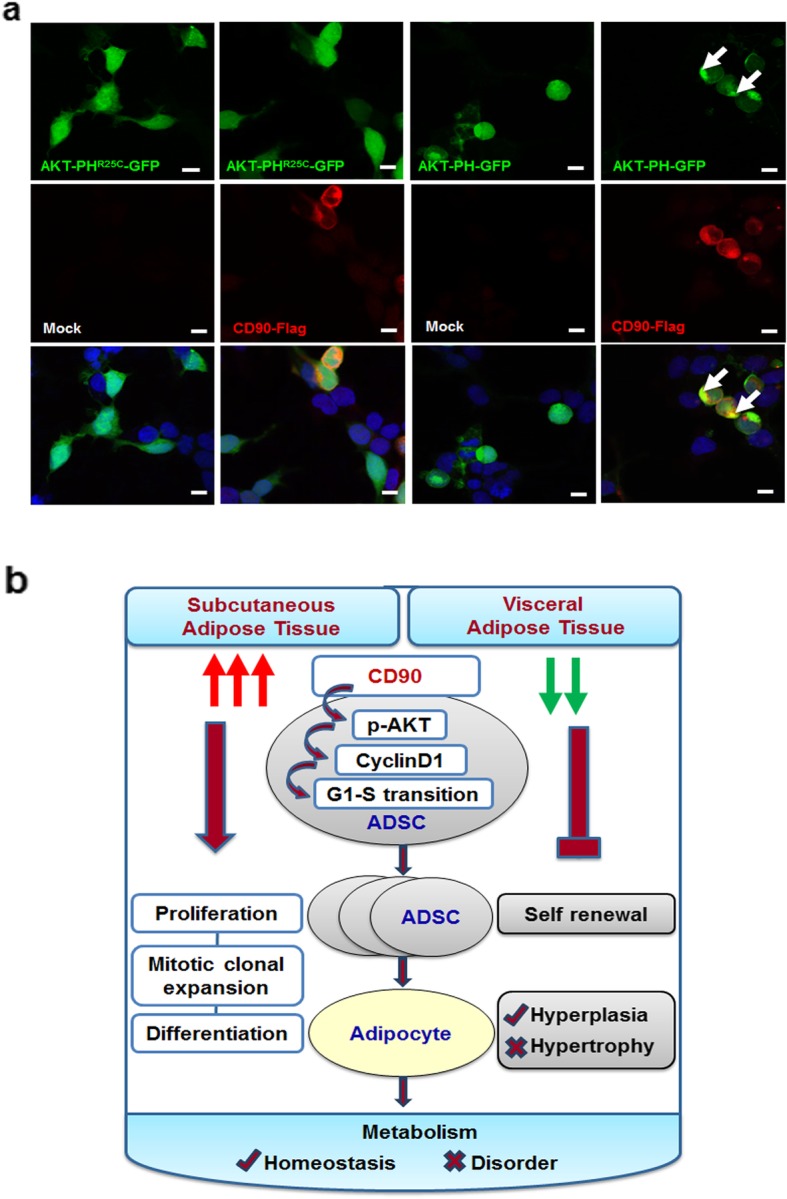
CD90 recruits AKT-PH domain to plasma membrane. **a** HEK-293T cells were co-transfected with pcDNA3-AKT-PH-GFP (or pcDNA3-AKT-PH^R25C^-GFP) and pENTER-CD90-Flag (or Mock). Fluorescence signals were detected under laser confocal microscope. Scale bar 20 μm. **b** A working model shows that CD90 differentially regulates ADSCs in SAT and VAT and metabolic homeostasis

The initial activation of AKT requires binding lipid messengers on plasma membrane with its active PH domain [47, 49, 50]. To clarify how CD90 regulated AKT activation, HEK-293T cells were co-transfected with pcDNA3-AKT-PH-GFP (or pcDNA3-AKT-PH^R25C^-GFP) and pENTER-CD90-Flag (or Mock), and the translocation of AKT-PH domain was detected. As shown in Fig. 8a, AKT-PH^R25C^-GFP was retained inside the cells and had no translocation to plasma membrane in either the presence or the absence of exogenous CD90-Flag, indicating that mutant AKT-PH^R25C^ domain loses the ability to bind to plasma membrane. A small amount of AKT-PH-GFP had a translocation to plasma membrane in the absence of exogenous CD90-Flag, indicating the basal level of endogenous CD90 may recruit AKT-PH domain in HEK-293T cells. Importantly, enhanced expression of CD90-Flag caused an obvious accumulation of AKT-PH-GFP in a raft-like structure, which had an apparent colocalization with CD90-Flag on plasma membrane, while AKT-PH-GFP inside the cells was accordingly reduced. Consistent with these observations, the overexpression of CD90 in ADSCs caused significant increases in AKT phosphorylation and CyclinD1 expression, which were obviously inhibited by MK2206 (Additional file 1: Figure S5). Thus, the overexpression of CD90 caused an obvious translocation of AKT-PH domain to plasma membrane, suggesting that CD90 promotes AKT activation by recruiting its PH domain to plasma membrane.

## Discussion

ADSCs play important roles in maintaining WAT homeostasis. The differences between S-ADSCs and V-ADSCs may influence the balance of hyperplasia and hypertrophy of adipocytes, which contributes to different metabolic profiles between SAT and VAT. In this study, we showed that S-ADSCs had higher proliferative potential than V-ADSCs, supported by increased expression of stemness genes *Nanog*, *Oct4*, and *Sox2*. Compared with V-ADSCs, S-ADSCs showed marked increases in AKT activation, CyclinD1 expression, and G1-S phase transition. Consistent with previous studies showing the critical role of AKT activation in promoting proliferation of cancer cells and ADSCs [45, 46, 51], we demonstrated that AKT activation in S-ADSCs may promote their proliferation via S phase entry driven by CyclinD1, thereby facilitating their self-renewal in SAT. Although there is still controversy over adipogenesis of SAT and VAT [18–20], our findings showed that S-ADSCs had higher efficiency in adipogenesis than V-ADSCs, indicating that S-ADSCs are more prone to adipocyte hyperplasia than V-ADSCs. Several recent studies showed that adipocyte differentiation was influenced by regulating mitotic clonal expansion, substantiating the indispensability of mitotic clonal expansion in adipogenesis [12, 26–28, 52, 53]. Our study provided evidences that S-ADSCs possessed high potential in mitotic clonal expansion, which may vest them with high capacity for adipogenic differentiation. Thus, S-ADSCs may exert important roles in maintaining WAT homeostasis due to their high potential of adipocyte hyperplasia.

Previous studies have reported that CD90 promoted the proliferation of hepatocellular carcinoma cells and hematopoietic stem cells, but inhibited the proliferation of ovarian cancer cells and nasopharyngeal carcinoma cells, or even displayed opposite effects on the proliferation of fibroblasts from different tissues [31, 33, 35, 36, 54–56]. Our study showed that CD90 was highly expressed on S-ADSCs but moderately on V-ADSCs. CD90 on S-ADSCs is essential for AKT activation and CyclinD1 upregulation, which may promote the proliferation and stemness of S-ADSCs via G1-S phase transition, and promote the mitotic clonal expansion of S-ADSCs and their terminal adipogenic differentiation. CD90 has been identified as a cancer stem cell marker involved in tumorigenicity in several types of cancers such as hepatocellular carcinoma [57–59]. By contrast, our findings revealed that CD90 on ADSCs served as an AKT activator to promote ADSC potentials and functions, suggesting CD90 may play beneficial or undesirable effects depending on its expression on different tissues or cells.

The potential link between CD90 engagement and activation of PI3K/AKT pathway was reported in human cytomegalovirus-infected cells, whereas the underlying mechanisms remain to be clarified [41]. The initiation of AKT activation requires binding to lipid messengers PtdIns(4,5)P2/PtdIns(3,4,5)P3 on plasma membrane through its active PH domain, which enables subsequent AKT phosphorylation [47, 49, 50, 60]. Our study showed the direct contribution of CD90 to AKT activation, as suggested by the translocation of AKT-PH domain from cytosol to plasma membrane caused by CD90 overexpression. Lipid rafts act as dynamic microdomains on plasma membrane and function in various membrane signaling pathways [61, 62]. Several studies have reported the critical roles of membrane raft microdomains in AKT activation [63–66]. As a GPI-anchored protein, CD90 has been verified to be incorporated into lipid raft and involved in signal transduction in various cells [62, 67, 68]. We showed that both exogenous AKT-PH domain and CD90 were exactly colocalized in a membrane raft-like structure, indicating that CD90 may act as an important trigger of membrane raft to promote the recruitment and translocation of AKT via PH domain, finally driving AKT phosphorylation and activation.

Based on its critical roles in ADSC proliferation and mitotic clonal expansion, CD90 exerts pivotal function in vivo in maintaining homeostasis of WAT and systemic metabolism. *CD90* silencing in inguinal SAT led to significant glucose intolerance in mice, though no significant alterations were observed in body weight and WAT weight. The metabolic change could be attributed to attenuations in ADSC self-renewal and adipocyte hyperplasia under physiological conditions, which eventually resulted in pathological adipocyte hypertrophy and consequent metabolic disorders. On some degree, this metabolic change can partially support a previous study showing increases in body weight and serum resistin level in CD90-null mice fed on high-fat diet [69]. However, different from the inhibitory effect of ectopic CD90 on the adipogenesis of 3T3-L1 cells, which influenced the whole process of adipogenic differentiation [69], we revealed an unrecognized role of CD90 in promoting mitotic clonal expansion, which influenced the early phase of adipogenic differentiation of ADSCs. Notably, although CD90 on S-ADSCs contributed to their proliferation and mitotic clonal expansion, a gradual loss of CD90 was observed during the process of adipogenic differentiation, which was consistent with the change of CD90 expression in differentiating 3T3-L1 cells [69, 70]. These data indicate that CD90 may play essential roles in maintaining the stemness of ADSCs, which determines their capacities for self-renewal and initiation of adipogenic differentiation in response to metabolic demands. After mitotic clonal expansion, the decrease of CD90 may drive the switch of ADSCs from proliferation toward terminal differentiation. Depending on the different expression levels, CD90 can differentially regulate the proliferation of S-ADSCs and V-ADSCs via AKT/CyclinD1 pathway, thus mediating distinct metabolic profiles of SAT and VAT. These findings were further supported by data from human and mouse WAT, as evidenced by the predominant expression of *CD90* in SAT rather than in VAT, and its positive correlation with AKT downstream *CyclinD1* but negative correlation with adipocyte hypertrophy-related *Leptin*. However, to validate the application potentials of CD90 in clinic, it is still necessary to expand animal or human samples in future investigations. It is also helpful to extend this study to gender differences, as both male and female have fat pads with different distributions and metabolic features. Furthermore, there was an increase of exogenous CD90 after blockade of AKT activation (Additional file 1: Figure S5), indicating a possible feedback regulation of CD90 by AKT signaling pathway in ADSCs or some influence of AKT signaling on the efficiencies of lentiviral infection or gene expression, which need to be further explored.

## Conclusions

This study provides evidences that CD90 is highly expressed on S-ADSCs rather than on V-ADSCs, which promotes AKT activation, CyclinD1 upregulation, and G1-S phase transition, thus empowering S-ADSCs with high potentials in proliferation, mitotic clonal expansion, and adipocyte differentiation. As consequence, high level of CD90 on S-ADSCs may contribute to metabolic homeostasis via preventing adipocyte hypertrophy in SAT, while VAT is prone to mediate metabolic disorder due to a reduction of CD90 on V-ADSCs (Fig. 8b). Therefore, CD90 acts not only as a valuable biomarker for evaluating ADSC potentials or monitoring metabolic status of WAT, but also as a potential target for treating obesity-associated metabolic disorders. It should be noted that more evidences are still required to elaborate the detailed functions of CD90 in ADSCs, WAT, and metabolism as well as the underlying mechanisms.

## Supplementary information

**Additional file 1: Table S1.** Sequences of siCD90 and shCD90. **Table S2.** Primer pairs used in qPCR. **Figure S1.** Inhibitory effect of *CD90* silencing on AKT activation. **Figure S2.** Infection of S-ADSCs with GFP-tagged shControl or shCD90 lentivirus. **Figure S3.** Influence of shCD90 lentivirus on WAT weight. **Figure S4.** Expression of *CyclinD1* and *Leptin* in adipose tissue. **Figure S5.** Contribution of CD90 overexpression to AKT activation in ADSCs.

## Data Availability

All data generated or analyzed during this study are included in this published article and its supplementary information files.

## Abbreviations

SAT: Subcutaneous adipose tissues
VAT: Visceral adipose tissues
ADSCs: Adipose-derived stem cells
WAT: White adipose tissue
S-ADSCs: Subcutaneous ADSCs
V-ADSCs: Visceral ADSCs
SVF: Stromal vascular fraction
MSC: Mesenchymal stem cell
GPI: Glycosylphosphatidylinositol
OD: Optical density
DMEM: Dulbecco’s modified Eagle’s medium
shCD90: CD90 shRNA
shControl: Control shRNA
FITC: Fluorescein isothiocyanate
PE: Phycoerythrin
PE-cy5: PE-cyanine5
GTT: Glucose tolerance test
H&E: Hematoxylin and eosin
PH: Pleckstrin homology
BSA: Bovine serum albumin
