# Supplementary material for: CD90 serves as differential modulator of subcutaneous and visceral adipose-derived stem cells by regulating AKT activation that influences adipose tissue and metabolic homeostasis
Source: Stem Cell Res Ther. 2019 Nov 28;10:355. doi: 10.1186/s13287-019-1459-7 (PMC6883612; doi:10.1186/s13287-019-1459-7)

**Additional file 1**

**Table S1. Sequences of siCD90 and shCD90**

| *siCD90-1* | Sense: CGAAUCCCAUGAGCUCCAAdTdT |
| --- | --- |
|  | Anti-sense: UUGGAGCUCAUGGGAUUCGdTdT |
| *siCD90-2* | Sense: GCCAUGAGAAUAACACCAAdTdT |
|  | Anti-sense: UUGGUGUUAUUCUCAUGGCdTdT |
| *siControl* | Sense: UUCUCCGAACGUGUCACGUTT |
|  | Anti-sense: ACGUGACACGUUCGGAGAATT |
| *shCD90* (GenePharma) | CGAAUCCCAUGAGCUCCAA |
| *shControl* (GenePharma) | TTCTCCGAACGTGTCACGT |
| *shCD90* (Genechem) | CGAATCCCATGAGCTCCAA |
| *shControl* (Genechem) | TTCTCCGAACGTGTCACGT |

**Table S2. Primer pairs used in qPCR**

| *Nanog* | Sense: CGGTGGCAGAAAAACCAGTG |
| --- | --- |
|  | Anti-sense: AAGGCTTCCAGATGCGTTCA |
| *Sox2* | Sense: GGCAAGGCAGAGAAGAGAGTG |
|  | Anti-sense: TCTGGCGGAGAATAGTTGGG |
| *Oct4* | Sense: TGATCCTCGAACCTGGCTA |
|  | Anti-sense: CTCAGGCTGCAAAGTCTCC |
| *CD90* | Sense: TGCTCTCAGTCTTGCAGGTG |
|  | Anti-sense: TGGATGGAGTTATCCTTGGTGTT |
| *C/EBPα* | Sense: TTGAAGCACAATCGATCCATCC |
|  | Anti-sense: GCACACTGCCATTGCACAAG |
| *Adiponectin* | Sense: CCTGTTCCTCTTAATCCTGCCCA |
|  | Anti-sense: ATCTCCTTTCTCTCCCTTCTCTCCA |
| *PPAR-γ* | Sense: GGAGCCTAAGTTTGAGTTTGCTGTG |
|  | Anti-sense: TGCAGCAGGTTGTCTTGGATG |
| *αP2* | Sense: GTGGGATGGAAAGTCGACCA |
|  | Anti-sense: ATCCAGGCCTCTTCCTTTGG |
| *Leptin* | Sense:TCAAGCAGTGCCTATCCAGAAAGTC |
|  | Anti-sense:GGGTGAAGCCCAGGAATGAAGTC |
| *GAPDH* | Sense: GTGTTTCCTCGTCCCGTAGA |
|  | Anti-sense: ATGAAGGGGTCGTTGATGGC |
| *18S rRNA* | Sense: GCCTGAGAAACGGCTACCACAT |
|  | Anti-sense: CCGCTCCCAAGATCCAACTACG |

**Figure S1.** **Inhibitory effect of *CD90* silencing on AKT activation.** S-ADSCs were transfected with siCD90 or siControl for 24 h, and stimulated with insulin (100nM) for different time periods. Protein levels of p-AKT, AKT were detected by western blot.


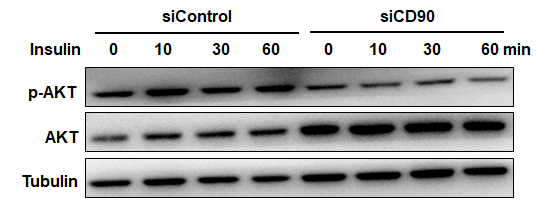


**Figure S2. Infection of S-ADSCs with GFP-tagged shControl or shCD90 lentivirus.** S-ADSCs were infected with GFP-tagged shControl or shCD90 lentivirus (GenePharma). The infection efficiency was determined by GFP signals under fluorescence microscope. BF, bright field. Scale bar = 100μm.


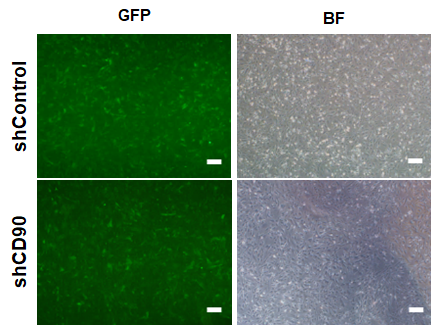


**Figure S3.** **Influence of shCD90 lentivirus on WAT weight.** GFP-tagged shControl or shCD90 lentivirus (Genechem) was injected into bilateral inguinal fat pads (7.0×10^6^ TU per point, 3 points per fat pad) of male mice at the age of 8 weeks, respectively. WAT weight was measured after 4 weeks of treatment.

**
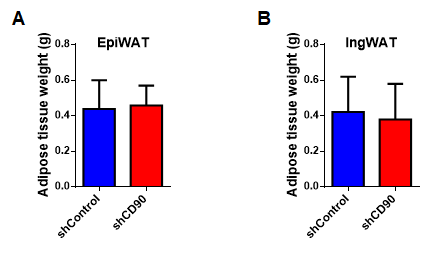
**

**Figure S4. Expression of *CyclinD1* and *Leptin* in WAT. A-C** The expression of *CD90* (A), *CyclinD1* (B) and *Leptin* (C) in inguinal, axillary SAT and epididymal, mesenteric VAT from mice fed on normal diet (GSE53307). **D,E** The expression of *CyclinD1* (D) and *Leptin* (E) in adipocytes and SVCs of epididymal VAT from mice fed on short-term of normal or high-fat diet (GSE65557). **F** The expression of *CyclinD1* in SAT and omental VAT from obese patients (GSE20950).

**
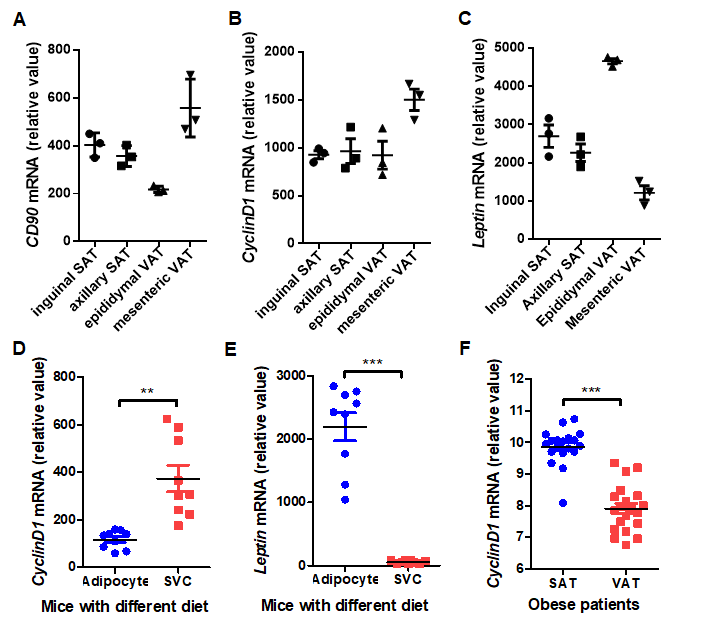
**

**Figure S5.** **Contribution of CD90 overexpression to AKT activation in ADSCs. A** CD90-Flag (Genechem) were overexpressed on ADSCs from human omental adipose tissue by lentiviral infection, protein levels of CD90-Flag, p-AKT, AKT and CyclinD1 were detected by western blot. Anti-Flag Ab was used to detect the expression of CD90-Flag. **B** ADSCs overexpressing CD90-Flag were incubated with MK2206 (2μM) for 16 h, protein levels of CD90-Flag, p-AKT, AKT and CyclinD1 were detected by western blot.


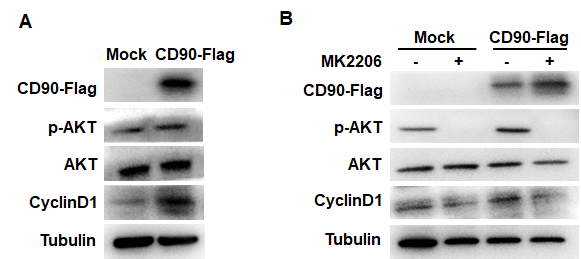

Supplement: Supplementary file 1 — Additional file 1: Table S1. Sequences of siCD90 and shCD90. Table S2. Primer pairs used in qPCR. Figure S1. Inhibitory effect of CD90 silencing on AKT activation. Figure S2. Infection of S-ADSCs with GFP-tagged shControl or shCD90 lentivirus. Figure S3. Influence of shCD90 lentivirus on WAT weight. Figure S4. Expression of CyclinD1 and Leptin in adipose tissue. Figure S5. Contribution of CD90 overexpression to AKT activation in ADSCs. [file 13287_2019_1459_MOESM1_ESM.docx]
